# Response to Comment on Jaworska, J. et al. Consensus on the Application of Lung Ultrasound in Pneumonia and Bronchiolitis in Children. *Diagnostics* 2020, *10*, 935

**DOI:** 10.3390/diagnostics11010066

**Published:** 2021-01-04

**Authors:** Anna Komorowska-Piotrowska, Joanna Jaworska, Andrzej Pomiećko, Jakub Wiśniewski, Mariusz Woźniak, Błażej Littwin, Magdalena Kryger, Piotr Kwaśniewicz, Józef Szczyrski, Katarzyna Kulińska-Szukalska, Natalia Buda, Zbigniew Doniec, Wojciech Kosiak

**Affiliations:** 1 Pediatric Pulmonology and Allergy Department, Medical University of Warsaw, 02-091 Warsaw, Poland; anna.komorowska-piotrowska@wum.edu.pl; 2 Cystic Fibrosis Department, Institute of Mother and Child, 01-211 Warsaw, Poland; joanna.jaworska@imid.med.pl; 3 Clinic of Pediatrics, Hematology and Oncology, University Clinical Center, 80-210 Gdańsk, Poland; apomiecko1@gmail.com (A.P.); wisniewski@gumed.edu.pl (J.W.); blittwin88@gmail.com (B.L.); magdalena.kryger@gmail.com (M.K.); jozefszczyrski@gmail.com (J.S.); 4 Department of Pulmonology, Institute of Tuberculosis and Lung Diseases, Regional Branch in Rabka Zdrój, 34-700 Rabka-Zdrój, Poland; wozniak.m.1986@gmail.com (M.W.); zdoniec@interia.pl (Z.D.); 5 Department of Diagnostic Imaging, Mother and Child Institute, 01-211 Warsaw, Poland; kwasniewiczp@gmail.com; 6 Pediatric Department of Respiratory Tract Disorders, Lung Diseases and Rehabilitation Center, 91-520 Łódź, Poland; sonokasia@dot.pl; 7 Department and Clinic of Internal Medicine, Connective Tissue Diseases and Geriatrics, Medical University of Gdańsk, 80-210 Gdansk, Poland; 8 Department of Pediatrics, Hematology and Oncology, Medical University of Gdansk, 80-210 Gdansk, Poland; kwojtek@gumed.edu.pl

Thank you for the opportunity to respond to the issues raised by Nenna et al. in the recent letter to the editor. All authors would also like to thank Dr Nenna and her colleagues for their recognition of the consensus [1]. We have read their recent research with great interest [2,3]. We share the enthusiasm regarding the use of lung ultrasound (LUS) as an excellent tool allowing to improve the management of lower respiratory tract diseases in children.

The timing of LUS performance is an interesting issue, regarding its impact on results and diagnostic value. The timeline of the baseline LUS examination may start relative either to the beginning of the symptoms or the beginning of the treatment. In the literature reviewed for the consensus, researchers presented a varying approach to this matter, with the first LUS examination being performed from up to 45 min [4] to 2–3 days [5] after hospital admission or within 2–15 days from the beginning of the symptoms [6]. The most commonly reported time frame was within the first 24 h after arriving at the hospital [7,8], which complies with the timing followed by La Regina et al. and Bloise et al. [2,3]. However, to the best of authors’ knowledge, there are no publications directly analyzing the impact of the time of the baseline examination on LUS findings, its sensitivity, and its specificity. Based on the articles published hitherto, it is still not possible to define the optimal timing for LUS performance in pneumonia and bronchiolitis. In clinical practice, it seems reasonable to perform the first examination as soon as possible.

The timing of the follow-up is another issue with far more differentiated timepoints. The first follow-up examination was performed in a time frame ranging from 48 h [9,10] through 3–6 days [11,12,13], 14 days [14] to 30 days [3]. All the publications reported LUS findings regression, which corresponded with clinical improvement. Taking into account all previously mentioned advantages of LUS, the method allows for numerous follow-up examinations relative to the patient’s clinical condition. The examination, performed 1 to 2 months after the treatment has been completed, allows for the detection of residual lesions, which may be especially helpful if a child develops another lower respiratory tract infection [1].

Both the literature and clinical practice prove the utility of LUS in diagnosing and monitoring different complications of pneumonia, including lung abscesses. The case described by Bloise et al. [3] reporting LUS detection of a small abscess cavity at an earlier stage than CXR, corresponds with our own clinical experience. Moreover, we also observe other complications (e.g., atelectasis) before they can be identified with CXR. However, it is worth highlighting that the localization of the lesion strongly impacts the value of LUS, especially in cases of lung abscesses.

Together with Nenna et al., we hope that following the already solid and still growing evidence, LUS will be implemented in the new guidelines on the management of pneumonia and bronchiolitis in the pediatric population.
